# Maternal Health Phone Line: Saving Women in Papua New Guinea

**DOI:** 10.3390/jpm5020120

**Published:** 2015-04-27

**Authors:** Amanda H.A. Watson, Gaius Sabumei, Glen Mola, Rick Iedema

**Affiliations:** 1 PNG Economic and Public Sector Program, PO Box 776, Port Moresby, NCD 111, Papua New Guinea; E-Mail: gaius.sabumei@gmail.com; 2 Visiting Fellow, School of International, Political and Strategic Studies, Australian National University, Canberra, ACT 0200, Australia; 3 School of Medicine and Health Sciences, University of Papua New Guinea, Port Moresby, NCD 111, Papua New Guinea; E-Mail: glenmola@dg.com.pg; 4 NSW Ministry of Health, Agency for Clinical Innovation, Faculty of Health, University of Tasmania, Hobart, TAS 7000, Australia; E-Mail: ram.iedema@gmail.com

**Keywords:** childbirth, communication, health communication, mHealth, maternal health, mobile phone, Pacific

## Abstract

This paper presents the findings of a research project which has involved the establishment of a maternal health phone line in Milne Bay Province of Papua New Guinea (PNG). Mobile phones and landline phones are key information and communication technologies (ICTs). This research study uses the “ICTs for healthcare development” model to ascertain benefits and barriers to the successful implementation of the Childbirth Emergency Phone. PNG has a very high maternal mortality rate. The “three stages of delay” typology was developed by Thaddeus and Maine to determine factors that might delay provision of appropriate medical treatment and hence increase risk of maternal death. The “three stages of delay” typology has been utilised in various developing countries and also in the present study. Research undertaken has involved semi-structured interviews with health workers, both in rural settings and in the labour ward in Alotau. Additional data has been gathered through focus groups with health workers, analysis of notes made during phone calls, interviews with women and community leaders, observations and field visits. One hundred percent of interviewees (n = 42) said the project helped to solve communication barriers between rural health workers and Alotau Provincial Hospital. Specific examples in which the phone line has helped to create positive health outcomes will be outlined in the paper, drawn from research interviews. The Childbirth Emergency Phone project has shown itself to play a critical role in enabling healthcare workers to address life-threatening childbirth complications. The project shows potential for rollout across PNG; potentially reducing maternal morbidity and maternal mortality rates by overcoming communication challenges.

## 1. Introduction

The maternal mortality rate in Papua New Guinea (PNG) is alarmingly high [1,2,3]. In fact, PNG has one of the highest maternal mortality rates in the world [4,5]. Rural health workers are often uncertain of what to do when birthing complications arise [6]. They typically have few support services and communication options. In addition, there is a severe shortage of health workers in PNG [7] and “an almost total lack of systematic in-service training, especially for rural health” [7] (p. xvi) (see also [8]).

Milne Bay Province (MBP) is one of 22 provinces in PNG. MBP has approximately 269,000 people [7,9], speaking about 48 languages. The capital of MBP is Alotau. MBP is known as a maritime province, as many coastal and island communities are not accessible by road. There are more than 600 islands, about 160 of which are inhabited. Mobile phone coverage reaches many parts of the province. There are 41 health centres, 147 aid posts and one hospital in MBP [9]. There are 788 health workers in MBP, of which 529 (or 67%) are government employees [7] and most of the remainder are employed by church-run health services.

This paper outlines the results of the first two phases of the Childbirth Emergency Phone project in Milne Bay Province (MBP). The project involved the establishment of a free-call phone number, which commenced operations on November 1, 2012. The phone line is available 24 h a day to health workers throughout MBP and is located in the labour ward of Alotau Provincial Hospital (APH). Health workers from around the province are able to call the phone for advice during childbirth emergencies. If an incoming call to the emergency phone is not answered, or if the phone line is busy, the incoming call is diverted to a second and, if necessary, a third number. The emergency phone is blocked for outgoing calls, in order to keep it free for incoming calls as often as possible. Follow-up calls or outgoing calls to medical staff are made through the hospital switch, which is operational 24 h a day. The hospital switch phone and the emergency phone sit next to each other in the labour ward.

### 1.1. Project Establishment

In a mobile phone project in Indonesia, it was found that perceived roles and “relationships among the different levels of healthcare workers could influence their use of” [10] (p. 359) technology in their work. Thus, organisational hierarchy could be a potential impediment to transfer of knowledge and collegial support amongst health staff. In the case of the current project, this potential impediment was a risk from the outset. A crucial early step in the process involved the management of the Milne Bay Provincial Health Authority (MBPHA) deciding that labour ward staff could give advice over the phone. This was a shift from the previous protocol of restricting advisory roles to medical officers. This decision was a crucial precursor to implementation of the project as there are frequent occasions when medical personnel are engaged in surgery or other tasks and cannot attend to requests promptly.

Labour ward staff received coaching and practised answering phone calls in role play simulations during the establishment phase of the project. During awareness sessions at rural health facilities, the project officer explained to staff members the specialised training and knowledge of midwives. Prior to this, the distinction between midwives and other nurses was not widely appreciated or understood. It is hoped that, over time, respect for and appreciation of midwives will grow, along with their skills and confidence in conveying appropriate advice and comforting support over the phone.

An important element of the project was visits to rural health centres by research staff and MBPHA staff to give rural-based staff the phone number and to explain the purpose of the phone line to them. An additional element of the project involved distribution of solar mobile phone chargers to rural health centres. Other materials provided to rural health centres included maternal health books, standard treatment manuals, stickers displaying the emergency phone number (very small stickers for placement on the reverse side of mobile phone handsets and larger stickers for placement on folders, medical kits and the like), and posters.

### 1.2. Phone Calls Received

The data in this section of the paper came from a logbook, into which incoming phone calls were noted by labour ward staff. In the labour ward, no staff member was assigned to answer all emergency calls coming in. Instead, calls were being answered by any labour ward staff member on duty. Labour ward staff were asked to note down all calls in a logbook. While staff may not have completed the logbook in every instance, there was evidence to show that at least 10 new cases, and usually more, were communicated using the phone line during each month (see Table 1).

**Table 1:** New and total number of calls recorded in the logbook.

Month	First Calls (New Obstetric Cases)	First Calls (New Non-Obstetric Cases)	Total Number of New Cases	Follow-Up Calls	Total Number Of All Calls
November 2012	17	2	19	49	**68**
December 2012	19	6	25	53	**78**
January 2013	27	2	29	22	**51**
February 2013	14	2	16	35	**51**
March 2013	27	5	32	51	**83**
April 2013	14	2	16	25	**41**
May 2013	10	4	14	17	**31**
**Total**	**128**	**23**	**151**	**252**	**403**

The free-call line was usually phoned by rural health workers located in rural health facilities where there was mobile network coverage. In most cases, they called to seek advice on maternal complications. The data suggests that the phone line became a key part of health service provision and demonstrates a continued need for such a phone line. The toll-free phone number was not disseminated to members of the public and was not being used often by members of the public, although there were some prank calls received.

## 2. Literature

### 2.1. Maternal Health in PNG

PNG has one of the highest maternal mortality rates in the world: 733 deaths per 100,000 live births [4,5,7]. It is sobering to note that the maternal mortality rate in PNG doubled between 1996 and 2006 [8]. In fact, “currently a woman in rural PNG has a one in 25 chance of dying in her lifetime as a result of childbirth” [4] (p. 28). For every maternal death, another 30 women sustain “significant disability, much of it life-lasting” [8] (p. vi). In addition, infant mortality is 57 deaths per 1000 live births [4,7]. There are “more women and children dying during birthing, than dying of malaria” [2] (p. 5). There are 12,300 births each year in MBP [6]. One assessment calculated the maternal mortality rate in a district of MBP to be equivalent to 787 deaths per 100,000 live births [11].

Maternal health is a complex problem requiring multi-faceted responses [8], such as family planning [8], training of midwives [3], reconstruction of dilapidated health centres and provision of emergency obstetric equipment [3]. Prior literature has emphasised the need for communication options to be available in all health facilities [8,11,12]. Communication is essential for “timely referral” [8] (p. xiv), which can make a crucial difference with respect to the number of women dying during labour [6]. Along with family planning and care of patients throughout pregnancy and delivery, provision of and access to emergency obstetric care is vital for saving lives [8].

### 2.2. Health Communication in PNG

The term “information and communication technologies” (ICTs) can include “the whole range of technologies designed to access, process and transmit information” [13] (p. 19). Mobile phones and landline phones are key ICTs. For years, communication regarding childbirth emergencies has remained challenging for health workers in MBP and throughout many areas of PNG. The installation of high frequency and very high frequency (HF/VHF) radios in rural health centres and hospitals began in 1999 in MBP and other parts of PNG [12] and enabled health centres with working radios to call referral hospitals about obstetric cases. In recent years, the usage of HF/VHF radio for emergencies and other medical reasons has decreased. A challenge of using radios is that confidential information is discussed in public spaces. In addition, most radios are not being serviced or repaired. Nonetheless, the radio network remains vital [12], particularly in places with no mobile phone coverage [11,12].

### 2.3. Mobile Phones and Healthcare

The use of mobile phones in healthcare is referred to as mHealth [14,15,16]. mHealth can be thought of as a type of eHealth, the latter being the convergence of Internet, computers and similar technologies in healthcare delivery [15]. mHealth is the utilisation of mobile telephony to advance “healthcare service delivery and improve beneficiary health” [14] (p. 69) (see also [17]). The term telemedicine is slightly different as it can include any form of phone but is typically limited to phone consultations between medical practitioners and patients [17], for example for follow-up after a patient has been discharged from hospital.

It has been argued that mHealth may render particularly promising benefits in developing nations [14]. PNG has been one of the last countries in the world to gain widespread mobile phone access, with network coverage extending to rural areas since the introduction of competition into the sector in 2007 [18] (see also [19]). This expansion of access to telephony across PNG has created opportunities for implementation of mHealth. The distribution of solar mobile phone chargers as part of the Childbirth Emergency Phone project was a strategy well-supported by previous research, which found that recharging mobile phone handset batteries is a challenging, and often costly, exercise in rural PNG [18,19].

mHealth is a nascent field [14,16]. Therefore, mHealth projects should all be complemented by thorough research [14] or appropriate evaluation [16,20]. Potential obstacles to mHealth success include “technical issues such as interference, power consumption, security, reliability, and interoperability” [17] (p. 30). Lessons gleaned from early mHealth projects suggest that the utilisation of mobile phones in the health sector can have positive impacts regarding “process improvements” [14] (p. 70) within the health system, although the evidence to date suggests “it is debatable whether improvements in system processes caused by introduction of mobile technologies translate into actual health outcomes” [14] (p. 72) for patients. Based on documented projects carried out to date, it appears that mHealth projects are more likely to succeed when they “extend or connect existing health resources by making human contact more efficient” [16] (p. 2). mHealth success increases if interventions are incorporated into existing health systems [16]. Project design should address “service quality, trust and situational factors” [15] (p. 44). In addition, mHealth projects are more likely to result in positive outcomes if they are “more personalised and interactive interventions and those aimed at increasing social support as well as knowledge” [16] (p. 2).

### 2.4. ICT4H Model

**Figure 1:**
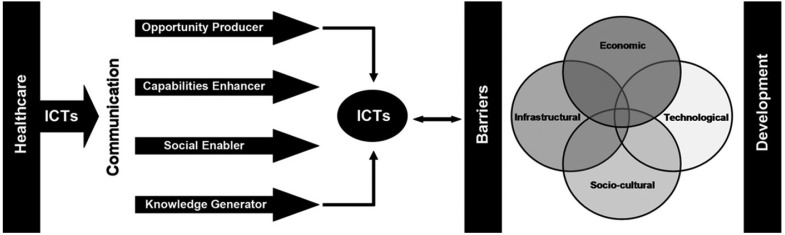
Information communication technologies (ICTs) for healthcare development model [10]. (Reproduced with permission from Taylor & Francis, Ltd., www.tandfonline.com on behalf of AMIC / SCI-NTU).

The “ICTs for healthcare development” (ICT4H) model designed by Chib *et al.* [10] (see Figure 1) presents potential benefits that ICTs can provide in a healthcare setting, whilst also considering possible barriers that may limit positive impacts.

The authors developed the ICT4H model based on an earlier model which presented anticipated benefits [21] but failed to outline potential barriers [14]. The left-hand-side of the figure indicates four benefits enabled by the use of ICTs in healthcare delivery. Chib *et al.* explain these benefits [10] (p. 350), as follows: ICTs could be seen as opportunity producers if they “facilitate work productivity” or increase the number of patients attended to; ICTs could enhance the capability of a health system to “make more timely referrals”; ICTs could enable social relationships “and professional engagement between healthcare workers”, and ICTs could generate knowledge “by improving access to medical information for healthcare workers”.

The right-hand-side of Figure 1 demonstrates four potential barriers to the effective implementation of ICTs. These barriers may act to limit the achievement of the benefits depicted on the left-hand-side of the diagram. The four barriers posed are inter-related [10] (p. 351), as follows: economic barriers to uptake or use of ICTs; infrastructural barriers such as limited “rollout of telecommunication networks, especially in remote areas”; socio-cultural inhibitors, such as those evidenced in “traditional values and practices” that may cause reluctance to utilise ICTs, and technological barriers, including difficulties with using ICTs stemming from “unfamiliarity and insufficient skills”.

The ICT4H model has been used as a theoretical framework for mHealth projects in the Asia-Pacific region, including: a maternal health project in Indonesia in which rural midwives were given mobile phones [10,14], a feasibility study regarding establishment of a toll-free maternal health line in Bangladesh [21], a study with rural doctors in China [22], and an evaluation of the use of mobile phones by rural community healthcare workers in India [23]. The model has been found to suit the context well in all the cited cases, although in the Bangladesh research the model’s suitability was implied rather than specified. One study has determined that the ICT4H model “can be critiqued for denying an active role to the agent, thus veering towards a form of technological determinism” [24] (p. 498) and the authors suggest a framework which allows participants to understand themselves [24].

Chib *et al.* studied a project in Indonesia in which rural midwives were given mobile phones and found that the new tools were deemed to be opportunity producers, as the mobile phones were used during emergencies not only as a vital communication tool, but also for arranging transport of patients [10]. As capability enhancers, midwives “believed that mobile phones enhanced their ability to handle medical situations” [10] (p. 356). Another key benefit of the Indonesian intervention was evidenced in an improvement of rural “midwives’ relationships with their colleagues and superiors in the healthcare hierarchy” [10] (p. 357). As knowledge generators, the mobile phones allowed midwives to improve their understanding by speaking to more experienced colleagues [10]. The four barriers shown in the ICT4H model were evident in the Indonesian case [10].

### 2.5. Three Stages of Delay

**Figure 2:**
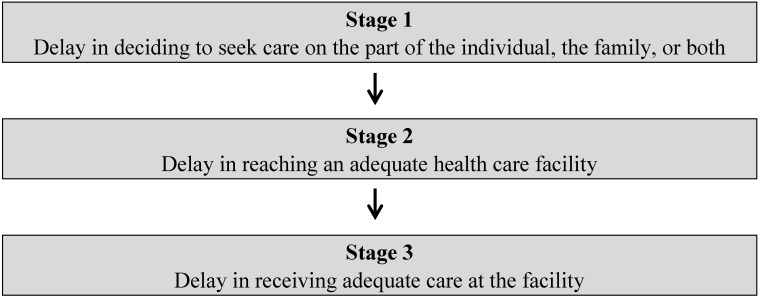
Three stages of delay (diagram based on [25]).

Complementing the ICT4H model, a second model, the three stages of delay, is used in this paper to analyse research findings. Thaddeus and Maine developed a typology of types of delays that may influence the health outcome of an individual case (see Figure 2). There may be multiple causes of a maternal death [8,25]. Roughly “75% of maternal deaths result from direct obstetric causes” [25] (p. 1092) and a majority of these deaths could be “prevented with timely medical treatment” [25] (p. 1092) (see also [26]). Therefore, any delay in the provision of appropriate medical treatment could be seen as a key catalyst of maternal death [25].

The model assumes that most women commence delivery at home, without a healthcare worker present [27]. In some cases, during a delivery in a village setting, a problem arises. The first type of delay is “estimated from the moment somebody … realised there was a problem until the decision to seek care was made” [6] (p. 57). This delay is influenced by various factors, such as the people involved in the decision-making process, the status of women in the particular setting, “previous experience with the health care system; and perceived quality of care” [25] (p. 1092). The second delay stage refers to the delay in reaching a suitable health care provider and is thus particularly affected by distance, transport availability, road condition, and so on [25]. The third type of delay occurs at a health facility and relates to the time taken for suitable care to be provided. Thus, the third delay stage can be influenced by factors such as “shortage of supplies, equipment, and trained personnel” [25] (p. 1092). In some cases, more than one type of delay may be involved, but delay experienced in any one stage could result in a maternal death [25].

The three stages of delay model has been used by Kirby to examine maternal deaths in MBP [6,26]. The review indicated that most maternal deaths are preventable and that delays are commonly experienced [6]. The value of promoting supervised deliveries through health promotion messages was emphasised [6]. Negative perceptions of health centres were mentioned in 22 out of 31 cases [6]. Viewing health workers as “unfriendly” [6] (p. 59) was also deemed to contribute to delays in seeking healthcare. Male dominance was an issue of concern [6]. Factors influencing the second and third types of delay included walking distance from a health facility [6], “lack of basic drugs and supplies” [6] (p. 58) and “lack of suitably trained staff” [6] (p. 58). The author also considered whether poor amenities for patients at health centres might be a deterrent: “most health facilities have poor washing and toilet facilities and lack decent waiting houses and, as such, are not mother-friendly places for confinement” [6] (p. 59).

In a study in Zambia, the model was utilised to examine why few women have supervised deliveries [28]. Factors decreasing the likelihood of a woman to have supervised deliveries were found to include “long distances, lack of transport, user fees, lack of adequate health education” [28] (p. 390) and a poor standard of service, staff and supplies at health facilities [28]. Gabrysch and Campbell suggest that the model is most suitable in contexts where the majority of births are unsupervised and therefore, in order to incorporate births occurring at health facilities, they have adjusted the model to distinguish between behaviours seeking emergency care as opposed to preventive care [27]. A recent review concentrated on the third delay stage and argued that “a focus on patient-side delays in the decision to seek care can conceal the fact that many health facilities in the developing world are still chronically under-resourced and unable to cope effectively with serious obstetric complications” [29] (p. 1). Poor referral communication was one issue raised in a review of literature on maternal mortality, “because of non-functioning radios and telephones or a complete lack thereof” [29] (p. 5).

## 3. Research Design

Research was undertaken in an ethical manner, by adherence to accepted standards of good practice in research ethics. Guiding principles which informed the research included honesty, integrity, respect for participants, and responsible communication of research results.

### 3.1. Research Questions

While the primary research question guided the research overall, the first two sub-questions related specifically to the ICT4H model. The third sub-question was referred to with particular attention to the three stages of delay model. The questions used were: 

Primary Research Question: Can the use of mobile phones and a free-call emergency number assist in improving maternal health outcomes and/or health system efficiency in Papua New Guinea?

Sub-Questions:

What are the benefits of mobile phone usage in the rural healthcare context, specifically opportunity production, capabilities enhancement, social enabling and knowledge generation?

What are the inter-related constraints to mobile phone usage in the rural healthcare context, specifically infrastructural, economic, technological, and socio-cultural factors?

Regarding communication systems, what are the key factors, benefits and barriers that contribute to, or detract from, healthcare system outcomes and health impacts?

### 3.2. Research Methods and Data Analysis

Semi-structured interviews with rural health workers in health centres and aid posts were undertaken over a 12-month-long period during visits to facilities. Interviews of recent mothers and village leaders were also completed during field trips. Labour ward staff were interviewed on two occasions: at the outset of the project and again a few months into the operation of the phone line. Table 2 shows the interview data included in this article.

Interview question schedules were prepared with a slightly different approach and focus, depending on the target group: rural health worker, labour ward staff member, recent mother or community leader. In the case of rural health workers, questioning commenced by ascertaining the respondent’s educational and work background, before discussing in detail their present work and then turning to maternal health and the health worker’s experiences with patients in childbirth. Questioning was designed to make the health worker feel comfortable and share their experiences. For example, one question used was: “Can you please share with me a story about a time when a woman went into labour and you were involved in this?” In the latter stages of interviews, rural health workers were asked for their thoughts on the Childbirth Emergency Phone, barriers that might limit its success and suggestions to help improve the project.

Labour ward staff members were interviewed in two rounds of five people. Three people were interviewed twice, while four people were interviewed only once. As with rural health workers, labour ward staff were asked about their background, their day-to-day work, their experiences with maternal health and finally their thoughts on the project. In the second round of interviews, labour ward staff members were asked to outline specific experiences related to the project. Recent mothers were asked about their background, their family, their experiences with health services and experiences of childbirth in their home village. Community leaders were asked about the village context, healthcare services in the area and community behaviours and attitudes regarding childbirth and maternal deaths.

Two focus groups were undertaken with rural health workers because in two particular cases they preferred to discuss as a team, rather than individually. Specifically, one focus group included three rural health workers, while the other consisted of six people: five rural health workers and an ambulance driver. Informal focus groups were also undertaken at key stages in the project with labour ward staff and the wider obstetric and gynaecological team, usually during weekly team meetings. Observations were made by the project officer, based in Alotau. The project office was located at APH, close to the labour ward, allowing for frequent engagement with staff, monitoring of the phone line at the labour ward and regular attendance at team meetings. The project officer also made field trips around MBP. Data was also gathered from a phone logbook in the labour ward (see Section 1.2).

All interviews and focus groups were recorded using a portable audio recorder and later transcribed. Analysis of qualitative data was undertaken by looking for emerging, regularly occurring or notable themes. Once key themes were identified as being of import for the interviewees, focus group participants or team members, these were further analysed with respect to the two theoretical models. Thus, the key concerns of research participants were weighed against the models, in order to determine the suitability of the models.

**Table 2:** Interviews conducted for the study.

Type of Interviewee	Number of Interviews
Rural health worker, based at health centre	25
Rural health worker, based at aid post	3
Labour ward staff member, round 1, 2012	5
Labour ward staff member, round 2, 2013	5
Mother	2
Community leader	2
**Total**	**42**

## 4. Results in Relation to Theoretical Models

### 4.1. Findings in Relation to ICT4H Model

During the 42 interviews conducted, all interviewees stated that the project solved the communication problems faced, especially when attending to obstetric emergencies. This point was also made by participants during the two focus groups held with rural health workers. The project was met with widespread enthusiasm, with both rural health workers and labour ward staff pleased to be able to communicate more effectively regarding childbirth complications. The project strengthened teamwork and improved communication between rural health workers and labour ward staff. As stated by a labour ward staff member, “things are now in order unlike before with the problem of under staffed … communication structure is flowing [more] easily than before”. Health workers saw the new project as helpful for them. Communication had been a major problem, as stated by all 42 interviewees. HF/VHF radio was the main mode of communication during emergencies before the expansion of mobile network coverage. Many of the health centres once had radios installed. However, most are not being serviced and repaired. The introduction of mobile phone service has increased the communication options between rural health centres and the labour ward. With network expansion, problems were encountered, as highlighted by a rural health worker as the project was commencing:

“Now we also have phones that we can be able to communicate out. But the only problem is sometimes phones too they don’t have units (mobile phone credit). Out here in the remote (areas), we find it difficult to find a unit. When we have a unit available, then we can be able to communicate out. And that’s when if we can be able to manage what we can then but like if we need the extra advice from the what (colleagues elsewhere), that’s the difficulties we are facing.”

In rural health facilities, clinicians were also pleased about the impact of the project, as expressed by one rural health worker:

“The project is very good. Before, I saw a mother die in front of me, because of no communication. Today, anywhere I can call, and it’s free. I don’t need to spend any money and it’s very good. I’m really happy and I thank the Australian government for this initiative to help people, especially those of us serving the remote, rural majority out here. They’re lowly educated, they do not have much money also, services are far away and we are the ones, we attend to them.”

The qualitative findings of the present study indicate synergies with the ICT4H model (see Figure 1). Wide-ranging benefits of the Childbirth Emergency Phone have been found to relate to all four benefits indicated in the ICT4H model. As an opportunity producer, the Childbirth Emergency Phone project provided numerous benefits, such as: providing a communication option at health centres where other forms of communication technology are not available, allowing health workers to phone the hospital even when they have no credit in their mobile phone, enabling more patients to benefit from the healthcare expertise within the province, and providing access to the best available level of midwifery advice while a patient is still located at a rural facility.

Such benefits reflect the facilitation of work productivity [10] enabled through the project. Health staff became able to support one another in attending to a greater number of patients, and in particular in addressing minor concerns before patient wellbeing deteriorated to become life-threatening and therefore highly resource intensive. All of these benefits were “thus creating an opportunity for increased monetary benefits for the healthcare provider” [10] (p. 350) as well as improved patient care and patient outcomes. One rural-based healthcare worker viewed the phone line as creating an opportunity for collegial discussion of cases: with evident respect for the knowledge, training and experience of labour ward midwives, the interviewee was pleased to be able to call in to seek a second opinion regarding problematic cases.

In terms of being a capability enhancer, the Childbirth Emergency Phone project allowed individual capabilities of health workers to improve and strengthened the capability of the healthcare system to “make more timely referrals to more advanced facilities” [10] (p. 350). In short, “the process of seeking for assistance” [10] (p. 356) from fellow health practitioners was made easier, quicker, less costly and more efficient. As a capability enhancer, the free-call number enabled health workers in MBP to feel more confident in handling cases and became helpful in providing timely support and coordinating transport of patients when necessary. As was mentioned by all interview respondents, the Childbirth Emergency Phone project helped to solve communication barriers between rural health workers and APH. The project also created positive outcomes in terms of providing better health service. It helped health workers in clinical work, for example in identifying common obstetric complications. As explained by one labour ward staff member, the labour ward staff were happy to be able to help rural health workers and their patients:

“With the phone coming up, you realise that we are all very happy, because now we can have calls straight coming into the ward and we can answer to their calls, especially their needing help, because we see the difficulty of the whole, you know, process of getting, I mean the whole process of communicating, for a patient, for an obstetric emergency.”

Indication of the Childbirth Emergency Phone project acting as a social enabler was found in qualitative interviews. Labour ward staff generally handled phone calls in a friendly and encouraging manner and staff felt like they were getting to know one another, thus allowing rural-based workers to feel at ease and able to openly discuss cases. The project helped in establishing good working relationships and collaboration between rural health workers and labour ward staff, as explained by a labour ward staff member: “rural clinicians are now getting to know labour ward staff which makes them at ease when seeking for advice and help.” Many clinicians felt they could be more open with each other, after getting to know one another, as a labour ward staff member said: “discussing cases over the phone with rural health workers makes us (feel we are) being part in treating the patient.” A labour ward staff member explained some of the benefits of the project: “the phone project is not just emergency but relationship within. We get to know more about what is happening out in the rural facilities”.

With regard to being seen as a knowledge generator, the Childbirth Emergency Phone project has provided examples of learning occurring at both ends of the communication link: rural-based healthcare workers were learning how to attend to certain types of cases and labour ward staff were learning more about the types of cases occurring. Learning and sharing of knowledge were discussed in the research interviews. Despite the learning that was taking place, repeated requests were made for training: 18 health staff (11 rural staff and seven labour ward staff) requested training for staff in both settings. While clinical knowledge and practice have changed over the years, for example with the availability of new drugs, some staff acknowledge they have not kept abreast of these developments.

There were many positive stories of how the project helped in assisting rural health workers with obstetric complications, and these stories often included reference to the sharing of knowledge during phone calls. A mother mentioned that “it’s a good project, to help the staff. That’s part of helping our health workers. When the health workers cannot make it to their end, they can get help from this project.” As told by a rural health worker, the phone service can generate knowledge and skills, while also helping to reduce social isolation and thus acting as a social enabler:

“Straight after we got this information about the free-call to the labour ward, I had this retained placenta. And I went. For three days, this mother was, you know, placenta was retained, and when I went to the clinic site, straight away from there I talked to the labour ward staff and I got advice on what to do. Had I not, you know, if this project was not here and I didn’t have any means to communicate, we could have lost this mother because she was already septicaemic. And so I find it very helpful. It’s very good. From anywhere, any point, I could seek advice. Very isolated or what, I could seek advice and I can help the patients.”

In the case of the Childbirth Emergency Phone project in MBP, economic barriers were largely removed due to the free-call nature of the phone line. Rural health workers expressed much enthusiasm about being able to phone the hospital for free. Previously, they were spending their own money to make work-related phone calls on their personal mobile phones [12]. Labour ward staff were also pleased to be saving money on phone calls, given how much money they were previously spending on work-related calls. Technological barriers were not significant as most health workers already owned mobile phones and knew how to make phone calls with them. Thus, barriers around technological literacy (the ability of an individual to operate a piece of technology [10]) were negligible.

Infrastructural barriers have impact in remote areas without electricity and/or mobile phone coverage. Distribution of solar mobile phone chargers helped to address the infrastructural barrier of poor electricity access, enabling rural health workers to recharge their mobile phone handset batteries. Out of the 42 interviews, 30 rural health workers were interviewed and although no question specifically addressed the distribution of solar mobile phone chargers, 10 interview participants mentioned they were very happy with the project distributing solar mobile phone chargers, saying it solved their problem of charging their mobile phone batteries, due to lack of electricity infrastructure: “communication would be alright because everybody is equipped with phones. If batteries are shut down then it would be a problem we would face” and “the portable solar charger is helping us. Thank you very much. You know, (there is) no power here, so this helps us”.

Communication challenges persist in places without network coverage, such as at several health centres in Samarai-Murua District. For staff at some health facilities, coverage is available nearby, but not at the health facility itself. For example, at Basima, staff have to paddle out to sea in a canoe to access mobile phone service. A disadvantage of this is that they are not with the patient when ringing and therefore cannot monitor patient vital signs or carry out instructions during a phone call. Another drawback is that follow-up calls from the labour ward will not reach staff if they are at Basima health centre.

Socio-cultural impediments were identified in a study of maternal deaths throughout MBP [6]. In the present study, socio-cultural barriers were also found, including traditional beliefs and knowledge levels. These barriers may decrease the likelihood of a delivery being supervised and therefore reduce the efficacy of the project as health workers are not at hand to identify problems if they begin to emerge. At present, it is not a common practice for men to attend antenatal clinics, maternal and child health clinics or family planning. Of the 42 interviews conducted, 12 interview respondents suggested that either a female relative or the woman’s husband should attend the delivery. The import of men’s roles in childbirth decisions has been noted in other studies [6,30].

### 4.2. Findings in Relation to Three Stages of Delay

**Figure 3:**
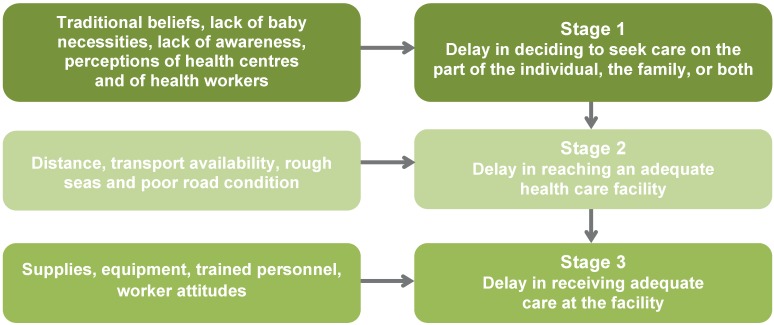
Factors contributing to the three stages of delay (diagram based on [25]).

Throughout the research interviews, instances of all three types of delay were evident. Factors contributing to these delays are shown in Figure 3. Regarding delays in the decision to seek care (Stage 1), there were five main contributing factors which emerged as recurrent themes: traditional beliefs and customs (15 out of 42 interviews), lack of baby necessities (15 out of 42), lack of awareness about the need for and value of supervised delivery (18 out of 42), community perceptions of health centres and community perceptions of healthcare workers. Factors listed in the literature as contributing to delays in reaching an adequate health care facility (Stage 2) were mentioned repeatedly in interviews, including distance, transport availability and poor road condition [25]. In keeping with Thaddeus and Maine’s assessment, factors contributing to delays in receiving adequate care at a facility (Stage 3) in MBP included lack of: adequate supplies, suitable equipment and trained personnel [25,28]. Some interviewees also suggested that poor attitudes of some healthcare workers may contribute to the third type of delay.

In the developing world, many pregnant women do not make it to health centres for supervised delivery because of a range of factors (Stage 1 and Stage 2 in the three stages of delay model). The issues reported here are the Stage 1 delays that were seen as being the most prominent concerns by interview respondents in this study. Eight interview respondents (*n* = 42) suggested that pregnant women and their families lacked awareness about the value of supervised deliveries. Interview respondents said that in some cases, women were not attending antenatal clinics, while in other cases they attended only one antenatal clinic during the pregnancy, and in still others they did attend antenatal clinics but then they delivered in the village. It was suggested that more health education and awareness activities should be conducted in order for people to learn about safe delivery. Fifteen interview respondents explained that some pregnant women felt shy because they did not have the items required for the delivery and the first days of nursing a baby, such as linen and other items. Some women were poor and could not provide for their babies, so they were ashamed to deliver at health facilities.

Fifteen interview respondents mentioned taboos, culture and traditions as barriers to safe delivery, with the following two quotes coming from rural health workers: “there are some taboos in some areas. Some mothers, as I am a male health worker, might feel too hesitant to approach me” and “they used to believe customs, traditions. They have that strong belief and they don’t want to come to the health centre for check-up. So, whatever they take in, such as bush herbs, sometimes it causes death”. While these issues are clearly linked to Stage 1 (delay in deciding to seek care), they are also socio-cultural barriers, as shown in the ICT4H model. A rural health worker described instances of socio-cultural barriers encountered:

“There are numerous number of them, for example, we have one girl, … she was at her about third trimester, I mean almost to deliver and then she got sick, not knowing that (her) mother … died from preeclampsia toxemia, nobody knew that she was a preeclampsia toxemia, she was undiagnosed preeclampsia toxemia, so when she had that high sudden fever and then her heart started to go faster she just passed away, here no one knew, she just slept and died, it was preeclampsia toxemia. But to them the parents, they said, not this one, they pwakau (poisoned), her already, they poison her or gave her something and she is like that, so to us is poor negligence or poor misdiagnose or other things but to them, village community is not that, it’s that thing, Poison. (…) Even, death that happens in the health centre has a traditional cause of death more than the physical illness itself, so in one other sense is that there is a need for awareness and health education in a sense that everyone must know that every deceased has a pathological cause or other socio-economical factor involve in it. Otherwise, this people will not understand yet.”

Regarding the third stage of delay (delay in receiving adequate care at a facility), there were a number of relevant issues raised during research interviews. Eighteen of the interviewees said that staff shortage was a major challenge at health facilities. There was some reference to a lack of training, skills and knowledge, as explained by a labour ward staff member: “sometimes they (rural health workers) are not confident to carry out some of the advices we are telling them.” Another issue that was raised during interviews was a lack of resources (medical drugs and equipment) at rural health facilities. As mentioned by two labour ward staff members: “their resources at health centres, on the other side of the line. They do not have the resources that might be needed to give that help to the mother” and “with the advice given over the phone, most of the rural health centres don’t have the resources and equipment to carry out the advice given”. An example case was provided by a labour ward staff member:

“I responded to a case this morning. Upon advice that the clinician should use oxytocin but it was out of stock. The patient was transported to the next health facility in order to execute the advice given. We here at the hospital have everything but I feel responsible when giving advice and the prescribed drug is unavailable. Every call we receive from rural health facilities, the patient becomes our patient and responsibility.”

In an earlier study undertaken in MBP, some of the same issues were seen as contributing to delays in women receiving adequate medical care for obstetric emergencies [6]. Traditional beliefs about placentas negatively impacted upon the decision to seek care [6]. The challenge of women not possessing linen and other items deemed necessary for birthing at a health facility was raised [6] and it is noteworthy that subsequent efforts by that study’s author to distribute “baby bundles” [6] (p. 59) containing such items were mentioned by most interviewees in the Esa’ala area as positively influencing the decision to have a supervised delivery. Lack of awareness of the importance of deliveries being supervised by a health worker was not specifically referred to in the previous study, but the author emphasised the value of health promotion messages about supervised deliveries [6]. While the prior study identified male dominance as an issue of concern [6], this was not a theme that emerged in the present study.

## 5. Discussion

### 5.1. Practical Implications of Research

Maternal mortality presents a multi-faceted challenge to the health system in PNG [8]. The Childbirth Emergency Phone addresses a critical area of concern: communication. Communication is vital, particularly for difficult cases. Communication can enable timely referral and access to emergency obstetric treatment. The project is having an impact and can be operated in partnership with other strategies which are also needed to reduce the maternal mortality rate in PNG.

The evidence suggests that the Childbirth Emergency Phone project has filled a communication gap and addressed a felt need amongst health workers in MBP. The analysis of interviews and other research data has led to the development of practical and policy implications. Key implications include: the importance of sustaining the phone line; the value of repairing and maintaining the HF/VHF radio network; the potential for rolling out similar free-call line resources across PNG; the importance of health centre visits to accompany such a roll-out, and the benefit of conducting thorough research in conjunction with innovative projects.

The HF/VHF radio network is vital for health communication in PNG and should be maintained [12]. It provides an essential communication option, particularly in places with no mobile phone coverage [11,12] or at times when the mobile phone network may be unreliable. While the value of the Childbirth Emergency Phone is clear, effort should be made to repair the radios around MBP that are currently inoperable. A fully functioning radio network would result in staff at some health centres having more than one communication option available to them. In other places, either the mobile phone or the HF/VHF radio will remain the sole method available for accessing advice, support or assistance.

A health workforce crisis in PNG has been identified [7] and efforts are being made to address this issue as a matter of urgency [3]. Out of 42 interviews conducted (38 of which were with health workers), 18 health workers mentioned staff shortage as a concern, both in rural areas and in the labour ward at APH. Despite some concerns amongst staff about labour ward staffing levels at APH, team members remain committed to answering phone calls whenever the toll-free number rings. Indeed, time spent discussing a case over the phone may reduce the likelihood of that case becoming more serious and requiring transfer in a critically ill condition and may thereby save staff time in the long-run. This is particularly true when rural staff identify problems early and call in for advice, thus reducing the likelihood of life-threatening complications such as postpartum haemorrhage.

### 5.2. Theoretical Implications of Research

mHealth is a relatively new field and the current project provides one of the very first examples of a well-documented mHealth project in PNG which has been complemented by rigorous research. It has been suggested that mHealth can enable improvement of processes within the health system, but that these benefits may not necessarily result in enhanced service for patients [14]. In this particular case, it is pleasing to note that the project not only assists and supports health workers, but is also resulting in demonstrable impact in terms of saving women’s lives.

mHealth projects are more likely to succeed when they “extend or connect existing health resources by making human contact more efficient” [16] (p. 2) and when they are incorporated into existing health systems [16]. The current project consists of both of these advantages. A close relationship with the MBPHA has been one of the project’s strengths throughout its duration, from initial consultation through to launching and throughout implementation.

Successful mHealth projects typically address “service quality, trust and situational factors” [15] (p. 44), are “more personalised and interactive interventions” [16] (p. 2) and incorporate social interaction and support between players, as well as enabling knowledge transfer [16]. All of these elements are evident in the current project. The project officer’s visits to health facilities throughout the province have engendered not only an understanding of the project but also, importantly, a sense of trust. Credit goes to the labour ward staff answering phone calls, as they provide friendly, supportive assistance to colleagues in rural locations.

The authors have found that the ICT4H model and the three stages of delay model have both been highly useful for analysing the research findings. In the case of the ICT4H model, all four benefits were described by interview participants. Therefore, the research has shown that all four benefits can be achieved through implementation of well planned, targeted ICT interventions in PNG or similar contexts. The project specifically addressed the economic barrier of the cost of phone calls and the infrastructural barrier of poor or non-existent electricity supply. Nonetheless, infrastructural barriers still hindered the project, in particular the lack of mobile network coverage at some health facilities. Technological barriers were not identified as significant in this case as the project was designed around the use of simple technology. Nonetheless, some socio-cultural barriers to success were identified during the research. The research suggests that use of simple technology, coupled with consideration of electricity access and user fees can ensure that interventions overcome most of the potential barriers to success.

The three stages of delay model was most helpful for analysing the qualitative interview data. All three types of delay were evident in the PNG context. The development of Figure 3 was a useful exercise for the authors as it focused attention on the key, recurring themes and divided these into each of the three stages of delay. It was interesting to note an overlap between the two theoretical models: the socio-cultural barriers identified in the ICT4H model were seen as contributing to the first stage of delay in the three stages of delay model.

### 5.3. Limitations of Research

The field research was conducted over a 12 month period, in one province of PNG. In all four districts of MBP, most health centres were visited, along with many aid posts. Differences between districts were noted, but common themes emerged from interviews, meaning that findings about the impact of the phone line during the time period were generalisable. Nonetheless, the relevance of the specific data for post-project reality cannot be assumed. It would be worthwhile for follow-up research to be conducted at a later date.

The research interview panel primarily consisted of health workers, as their perspectives were of paramount importance for the design, operation and review of the project. Additional insights may be gained from speaking to a greater number of patients and community members. It was difficult to coordinate research visits to coincide with antenatal clinics, maternal and child health clinics and outreach patrols; attendance on these days may provide additional information.

## 6. Conclusions

Mobile phones are portable, easy-to-use, and allow health workers to access help and support if they are located within network coverage, including when they are out on patrols or if they need to attend to a patient at their home or while travelling. Mobile phones are relatively cheap to purchase and can be readily at hand. Mobile phones enable private and confidential discussion of cases. Mobile phone usage has typically incurred costs for individual health workers [12], although some health managers have attempted to provide mobile phone credit to staff.

The Childbirth Emergency Phone project is helping rural health workers. They are calling in to get advice on obstetric cases. The phone is mainly used by staff at rural health facilities where there is mobile network coverage. In most cases, they are calling in to seek advice on maternal complications. Advice is given during phone calls by staff in the labour ward at APH. Both rural health workers and labour ward staff have expressed enthusiasm about the toll-free phone line and its role in their work.

During awareness sessions conducted as part of the project, rural-based healthcare workers were encouraged to phone early, for example when at-risk mothers were identified during pregnancy, or as soon as a patient in labour showed some abnormal signs, rather than waiting until the patient’s condition deteriorated so as to become life-threatening. When patient transfer was deemed necessary, the phone line played a crucial role in assisting with techniques for stabilising the patient before the commencement of the journey. During research interviews, respondents provided examples of cases when the lives of a woman and/or her baby were saved through use of the phone line.

The findings of the present study suggest that the Childbirth Emergency Phone project has enabled healthcare workers to address life-threatening childbirth complications. The project shows potential for rollout across PNG; potentially reducing maternal morbidity and maternal mortality rates by overcoming communication challenges. Further research in MBP is recommended at a later date to determine if there are any changes in uses of the phone line over time.

The conduct of similar research in other provinces of PNG could also yield informative insights. Given the rich cultural, linguistic, topographical and contextual diversity across PNG [31], a strong argument could be made for studying local conditions and designing telephony approaches that enhance health service delivery for local communities. Therefore, it could be appropriate to trial phone lines within the health system in a small, select number of provinces, with reference to relevant local factors and complemented with rigorous research. A systematic approach to running trials and sharing the lessons learnt would help to determine efficacy in different contexts. Ultimately, this would allow for the most appropriate use of telephony within the health system country-wide, for the benefit of the people of PNG.
